# A Comparison of Cholesterol Uptake and Storage in Inflammatory and Noninflammatory Breast Cancer Cells

**DOI:** 10.1155/2012/412581

**Published:** 2012-12-31

**Authors:** Breonna J. Martin, Kenneth L. van Golen

**Affiliations:** Department of Biological Sciences and The Center for Translational Cancer Research, The University of Delaware, 320 Wolf Hall, Newark, DE 19716, USA

## Abstract

Although there are many subtypes of breast cancer, inflammatory breast cancer (IBC) is arguably the deadliest. Research over the past decade has demonstrated that IBC is a distinct entity from other forms of breast cancer. Important risk factors that have been associated with the development of aggressive breast cancers, such as IBC, include obesity and diet, which are evident in the United States, where the overconsumption of high-fat foods continues to contribute to obesity in the nation. Here we investigate differences in cholesterol uptake and storage between IBC, non-IBC, and mammary epithelial cell lines. Our results demonstrate that compared with human mammary epithelial cells (HMECs), both IBC and non-IBC cells have increased cholesterol content. IBC cells retain intracellular cholesterol esters, free cholesterol, and triglycerides in lipid-deficient environments. In contrast, we observe in cell-type-of-origin-matched non-IBC a significant decrease in lipid content under the same lipid-deficient conditions. These data suggest that cholesterol storage may be affected by the cholesterol content of the environment where the tumor cell was isolated. Here, we suggest that breast cancer cells may migrate when they are unable to obtain cholesterol from their extracellular environments.

## 1. Introduction

Breast cancer affects approximately 1 in 8 women making it the most commonly occurring cancer in women in the United States [1]. Inflammatory Breast Cancer (IBC), a particularly lethal subset of locally advanced breast cancer, is currently estimated to affect up to 6% of breast cancer patients in the United States [2]. While most non-IBCs are detected by the presence of a dense mass in the mammary tissue, IBC is characterized by rapidly progressing primary skin changes such as erythema, skin thickening, peau d'aurange, and nipple retraction [2, 3]. The unique appearance of IBC is due to tumor emboli that readily metastasize into and block the dermal lymphatic vessels of the skin overlying the breast [4]. The highly aggressive and metastatic nature of IBC contributes to the low 3-year disease-free survival rate of less than 40%, as compared to approximately 90% for non-IBCs [5]. IBC is also molecularly distinct from non-IBCs, demonstrating an overall difference in gene expression profiles compared to stage and/or cell-type-of-origin matched cancers (i.e., Luminal, Her2+, or basal gene cluster subtype) [6, 7]. Differences in expression of molecules associated with progression such as E-cadherin or caveolins have opposite trends in IBC versus non-IBC (reviewed in [8]). These differences present significant challenges in treating this aggressive disease with traditional breast cancer therapies. In order to identify innovative and effective treatment plans for both IBC and non-IBCs it is important to further our understanding of cellular characteristics that distinguish the two diseases from each other.

 Studies suggest that cholesterol and cancer are closely associated, where cholesterol tends to accumulate in the cells comprising solid tumors [9–12]. The mechanisms of cholesterol homeostasis are often dysregulated in tumors so that cholesterol deposition is favored [13, 14]. These findings are particularly interesting in the context of developed countries such as the United States where diets that are high in cholesterol and fatty acids are thought to be associated with a higher incidence of breast cancer [13, 15, 16]. Currently, the relationship between cholesterol accumulation and cancer progression is poorly understood. 

 In normal nonmalignant tissues intracellular cholesterol is closely monitored and adjusted to maintain appropriate cholesterol levels [17–20]. The accumulation of cholesterol that is often seen in breast cancer is most likely due to alterations in cholesterol acquisition, efflux and/or transport within the breast cancer cells. To gain further insight of which aspects of cholesterol regulation are altered, we studied a number of genes that are involved in such mechanisms: 3-hydroxy-3-methylglutaryl-CoA reductase (HMGCR) is the rate-limiting enzyme in the mevalonate pathway responsible for *de novo* cholesterol synthesis when intracellular sterol levels are low [21]. Low-density lipoprotein receptor (LDL-R) and scavenger receptor class B type I (SR-BI) increases intracellular cholesterol by facilitating lipoprotein transport from the extracellular environment [22, 23]. LXR*α* is a transcription factor that is sensitive to high intracellular cholesterol levels, and stimulates transcription of ATP-binding cassette, subfamily A, member 1 (ABCA1) [20]. ABCA1 is responsible for retrograde movement of free cholesterol from the cell and works by transferring free cholesterol molecules to acceptor proteins such as Apolipoprotein E (ApoE) [24]. Although there are many proteins involved in modulating intracellular cholesterol, these proteins are central in regulating cellular cholesterol levels [17, 19, 25, 26]. 

Since IBC is distinct from other forms of breast cancer, we sought to determine if there were differences in accumulation, metabolism, and utilization of cholesterol compared to non-IBC cells. In this study we compared the highly invasive SUM149 IBC cell line with the triple negative basal cell-type-of-origin matched, MDA-MB-231 non-IBC cell line [27]. Approximately 1/3 of IBCs are triple negative and the SUM149 cell line has proven to be an accurate representative of what is observed in triple negative patient IBCs [8, 28]. Together these cell lines represent IBC and non-IBC that are matched by gene cluster analysis, thereby reducing the probability that any observed differences in cholesterol handling be due to inherent molecular differences such as estrogen/progesterone receptor or Her2+ expression. We also included the MCF10a nontumorigenic human mammary epithelial cell (HMEC) line to compare with both tumor lines. Here, we investigated lipid levels at baseline and after cholesterol loading and depletion, expression of genes centrally involved in cholesterol homeostasis, and the effect of cholesterol availability on breast cancer cell invasion. We show that the IBC and non-IBC cells initially contain a large amount of stored cholesterol in the form of lipid droplets, whereas the nontumorigenic cells do not. We also show that the non-IBC cells are reliant on extracellular cholesterol levels to maintain intracellular stores, while the IBC cells are capable of maintaining intracellular cholesterol relatively independent of extracellular cholesterol levels. Furthermore, we show that the lack of extracellular cholesterol tends to drive breast cancer cell invasion, *in vitro*.

## 2. Materials and Methods

### 2.1. Cell Lines

All cell lines were verified for authenticity by the Johns Hopkins Genetics Resource Core Facility. SUM149 cells were maintained in Ham's F-12 medium (Mediatech) supplemented with 5% (v/v) FBS (Atlanta Biologicals), 1% L-Glutamine, 1% Penicillin/Streptomycin, 1% Antibiotic/Antimycotic, 1% Insulin/Transferrin/Selenium cocktail (all from Mediatech), and 1 *μ*g/mL Hydrocortizone (Sigma-Aldrich) as previously described [29]. MDA-MB-231 cells were grown in DMEM medium (Mediatech), supplemented with 5% FBS, 1% Penicillin/Streptomycin, and 750 ug/mL Insulin (Sigma-Aldrich). MCF10a cells were maintained in 50 : 50 DMEM/F-12 medium (Mediatech) supplemented with 5% FBS, 5 *μ*g/mL Insulin, 0.5 *μ*g/mL Hydrocortisone, 50 *μ*g/mL Bovine Pituitary Extract (Gibco), 20 ng/mL EGF (Sigma-Aldrich), and 100 ng/mL Cholera Toxin (Sigma-Aldrich). Cells were maintained in a tissue culture incubator at 37°C and 5% CO_2_ atmosphere.

### 2.2. Preparation of Low-Density Lipoprotein (LDL) and Lipoprotein Deficient Serum (LPDS)

Fresh human serum was generously donated from the Blood Bank of Delmarva (Wilmington, DE) for the purpose of LDL isolation. The density of the serum was adjusted to 1.063 g/mL using granulated NaBr (Fisher Scientific), and ultracentrifuged at 50,000 rpm for 22 h at 15°C. Ultracentrifugation was performed using a Beckman SW 60 Ti rotor (Beckman Coulter), and Beckman Quick-seal Polyallomer tubes (Beckman Coulter). The top layer was removed and density adjusted to 1.3 g/mL with NaBr. Density gradients were prepared inside of ultracentrifuge tubes with a top layer of ddH_2_O (*d*  =  1.0 g/mL), a middle layer of a NaBr solution (*d*  =  1.125 g/mL), and a bottom layer of lipoprotein solution (*d*  =  1.3 g/mL). The gradient was separated by ultracentrifugation at 50,000 rpm for 60 min at 15°C. After distinct separation of very low-density lipoprotein (vLDL) and LDL was visible, LDL was carefully removed and dialyzed against three changes of cation-free 1x PBS with 0.01% EDTA pH 7.4 at 4°C, and one change of cation-free 1x PBS at 4°C. The integrity of the isolated LDL was confirmed by gel electrophoresis, on a 4–25% polyacrylamide gradient gel. For storage, LDL was aliquoted and frozen in 10% sucrose buffer [30]. Before cell treatment, thawed LDL was dialyzed against three changes of 1x PBS with 0.01% EDTA (pH 7.4), and sterile filtered using a 0.22 *μ*m syringe filter (Thermo Scientific Nalgene). The concentration of LDL was determined using a BCA protein assay kit (Thermo Scientific).

Lipoprotein deficient serum (LPDS) was prepared from FBS (Atlanta Biologicals), using a previously described protocol [31]. The FBS was density-adjusted to 1.21 g/mL using granulated NaBr, and centrifuged for 48 h at 10°C at 22,000 ×g using a SW 41.Ti rotor (Beckman Coulter). After centrifugation, the top layer of lipoproteins was carefully removed beyond the noticeable gradient. The remaining LPDS was dialyzed in 4 changes of 1x PBS pH 7.4 at 4°C over the course of 24 h. LPDS was then sterile-filtered using a 0.22 *μ*m filter, aliquoted, and stored at −20°C until use.

### 2.3. Incubation in FBS, LPDS, and LDL

To standardize culture conditions for experiments, controls were performed using each cell line's respective base media and 5% FBS, with no other additive. Cells were washed with 1x PBS pH 7.4 and incubated in FBS-medium for 24 h prior to each experiment. For LPDS treatments, media containing 5% LPDS was prepared for each cell line in their respective base media. Cells were washed with 1x PBS before introduction of LPDS-medium. Cells were then incubated in LPDS-medium for 24 h before the start of individual experiments. For LDL treatments, cells were washed with 1x PBS and incubated in 5% LPDS-medium for 24 h. After 24 h in LPDS, LDL was added at a concentration of 200 *μ*g/mL of media, which corresponded to a physiologically relevant amount of 100 *μ*g/mL cholesterol. Cells were incubated in LPDS-media containing LDL for 12 h prior to each experiment.

### 2.4. Oil Red O Staining and Imaging

A main stock solution of Oil Red O (ORO) was prepared by dissolving 0.35 g Oil Red O powder (Sigma-Aldrich) in 100 mL isopropanol (Sigma). Before staining, a 0.2% Oil Red O working stock solution was prepared using 60% of the main stock solution and 40% ddH_2_O, and filtering with a 0.22 *μ*m syringe filter. Cells were washed with ice-cold 1x PBS (pH 7.4), fixed for 10 min with 4% paraformaldehyde in PBS, stained with 0.2% Oil Red O for 15 min, washed with ddH_2_O for 1 min, and allowed to dry. Images were captured using a Nikon TMS inverted phase microscope. Duplicate images were taken using phase contrast to visualize and count the number of cells in each image, and brighfield to quantify the staining. Bright field images were analyzed using ImageJ. Samples were compared using Analysis of Variance (ANOVA) followed by Tukey's test. 

### 2.5. Filipin Staining

Cells were plated in the four center wells of 8-well Lab-Tek II Chambered Coverglass overnight. Cells were washed 3 times in 1x PBS, pH 7.4, fixed in 4% paraformaldehyde (Electron Microscopy Sciences) in PBS for 30 min, washed again 3x in PBS, and incubated with 200 *μ*g/mL Filipin solution in 10% BSA in PBS for 1 h. Finally, cells were rinsed 3x in PBS, and stored at 4°C with a drop of SlowFade Antifade reagent (Invitrogen), in PBS for no more than two hours before imaging. Cells were imaged using a Highspeed/Spectral Confocal Microscope: Zeiss 5 LIVE DUO.

### 2.6. Quantitative RT-PCR

Total RNA was isolated from cells using RNeasy Mini Kit (Qiagen) according to the manufacture's protocol. Total RNA was DNase treated using an Ambion DNA Kit (Ambion) according to the manufacturer's instructions. RNA quality and concentration was analyzed spectrophotometrically. RNA (1 *μ*g) was then reverse transcribed using Oligo (dT) Primer (Ambion), 10 mM dNTP Mix (Promega), and M-MLV Reverse Transcriptase (Invitrogen) according to the manufacturer's instructions.

Real-time PCR experiments were carried out on reverse transcribed cDNA using primers (Table 1) at a final concentration of 0.4 *μ*M, and iTaq SYBR Green Supermix with ROX (BioRad) according to the manufacturer's instructions. Each sample was plated in triplicate wells, and amplification was carried out in an Applied Biosystems 7300 Real-Time PCR System. Amplification efficiencies were calculated for each primer pair and primers with less than 90% efficiency were excluded from final results. B2M, a gene expressed at similar levels of our proteins of interest, and minimally variant among breast cancer samples, was used as an internal control [32]. Real-time PCR analysis was quantified using the 2^−ΔΔCT^ method [33]. The results are reported as fold changes relative to FBS control cDNA for each cell line, after normalization to B2M internal control [32].

### 2.7. Immunoblotting

Western blot analysis was performed as previously described [34]. RIPA buffer with 10 *μ*L/mL phosphatase inhibitor (Thermo Scientific), and 5 *μ*L/mL protease inhibitor cocktail (Calbiochem) was used to harvest protein from each cell line. Protein lysates (30 *μ*g) were separated by SDS-PAGE on a 12% gel, transferred to nitrocellulose, blocked using 5% milk (BioRad) in 1x PBST overnight at 4°C. LDL-R and *β*-actin primary antibodies (Abcam and Cell Signaling Technology) were used at a dilution of 1 : 300 and 1 : 1000, respectively. After incubation with donkey anti-rabbit-horseradish peroxidase-linked secondary antibody, the protein bands were visualized using Immobilon Western Chemiluminescent HRP Substrate (Milipore), and X-Ray film. The developed film was scanned using a trans-luminescent scanner. Densitometry analysis was performed on the scanned images using ImageJ.

### 2.8. Invasion Assay

Invasion assays were performed using BD Matrigel Invasion Chamber 24-well plates (BD Biosciences). Cells were treated, 24 h before invasion assay was plated, with LPDS, LDL in LPDS, 5 *μ*M Atorvastatin in LPDS, or left untreated in complete serum-containing media. Cells were then washed, trypsinized, and seeded on Matrigel-coated filters (8 *μ*m pore) at a density 10,000 cells/mL in serum-free medium. Medium containing 15% FBS was used as a chemoattractant. For atorvastatin-treated cells, 5 *μ*M Atorvastatin was added to cells in the invasion assay for a total treatment time of 46 h. Cells were allowed to incubate for 22 h in a tissue culture incubator at 37°C and 5% CO_2_ atmosphere. After incubation period, noninvaded cells were removed from the filter using a moist cotton swab. The invaded cells were stained with 0.5% crystal violet solution containing 20% methanol, washed with distilled water, and allowed to dry for 24 h. The total number of invaded cells for each well was counted. 

### 2.9. Data Analysis

*In vitro* data were analyzed using a GraphPad software package for Windows (Prism 4.0). A *P* ≤ 0.05 was considered statistically significant. Experiments were performed in triplicate with multiple replicates per experiments.

## 3. Results

### 3.1. Breast Cancer Cells Have High Levels of Intracellular Neutral Lipids

**Figure 1:**
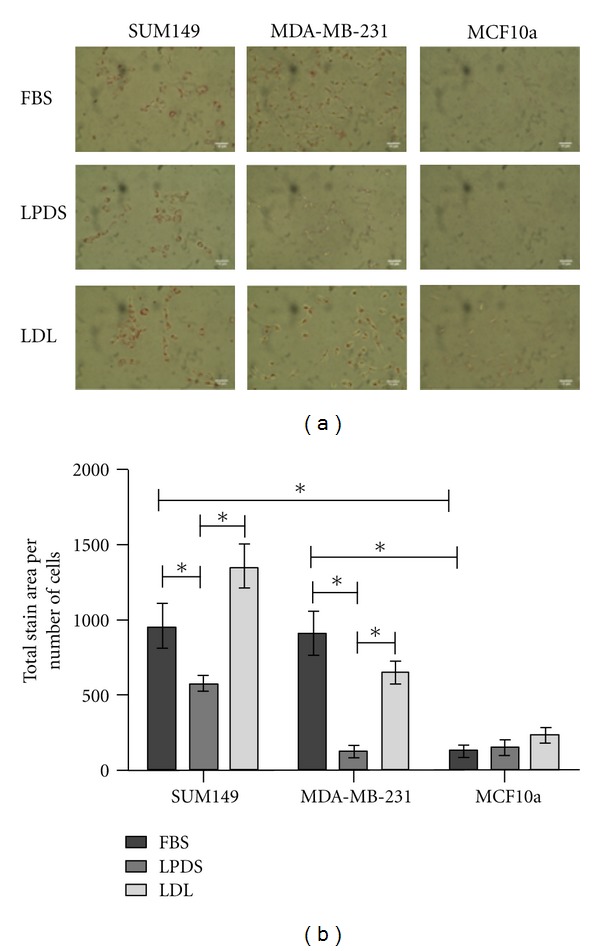
Oil Red O (ORO) staining in cells (SUM149, MDA-MB-231, and MCF10a) with FBS control, and treated with LPDS and LDL. (a) Representative bright field images for each treatment. The scale bars represent 10 *μ*m. (b) Quantitation of relative stain intensity (total stain area/number of cells) was performed for each image using ImageJ. Statistical significance (*P < 0.01) was determined by one-way ANOVA followed by Tukey's HSD test. The error bars represent SEM. All experiments were performed at least three separate times.

To determine baseline lipid levels, as well as the effect of lipid depletion and reloading on each breast cancer cell line, we performed Oil Red O (ORO) staining. ORO staining gives an indication of cellular cholesterol, cholesterol esters and triglyceride levels, which are referred to as neutral lipids. Representative bright field images of each cell line grown in medium containing FBS as an initial control are shown in Figure 1(a). The SUM149 and MDA-MB-231 cells lines display a significantly higher level of ORO staining as compared to the MCF10a cells. To demonstrate that MCF10a cells were present during ORO staining, a representative phase contrast image has been provided in Supplemental Figure 1 (see Supplementary material available online at doi:10.1155/2012/412581). As determined by densitometry of ORO staining, the SUM149 and MDA-MB-231 display significantly higher neutral lipid levels as compared to the MCF10a cells (Figure 1(b)). Similar results were seen for the triple negative basal cell-type-of-origin matched MDA-MB-435 cell line (data not shown). The results of the FBS control ORO staining suggest that the malignant cell lines initially contain a significantly larger amount of neutral lipid droplets, containing cholesterol, cholesterol esters, and triglycerides, than the noncancerous cell line. These observations are consistent with published data describing increased cholesterol content in breast cancer tumor tissues [12, 35, 36].

To analyze the baseline overall content and distribution of free cholesterol within the cells, we performed Filipin staining to visualize free cholesterol and aid in determining the amount of unesterified cholesterol present in cell membranes. MCF10a cells stain with the highest relative Filipin intensity, while MDA-MB-231 and SUM149 cells show significantly lower Filipin staining (Supplemental Figure 2). These data suggest that cholesterol in the HMECs are not stored but utilized in membranes.

Growth in medium containing lipoprotein deficient serum (LPDS) was used to determine the effect of a lipid-depleted environment on each cell line, while addition of low-density lipoprotein (LDL) was used to determine the effect of a cholesterol-rich extracellular environment on each cell line. In these experiments cells were grown in LPDS and then continued in LPDS +/−LDL added to the culture. ORO staining of SUM149 and the MDA-MB-231 cells grown in LPDS +/−LDL showed significant differences in neutral lipid content in response to changes in extracellular cholesterol. Incubation in LPDS-medium led to a significant decrease of intracellular neutral lipids in both the SUM149 and MDA-MB-231 cells (Figures 1(a) and 1(b)). Differences in ORO staining was most dramatic in the MDA-MB-231 cells after incubating in LPDS for 24 h, showing an 8-fold decrease in neutral lipid content. LPDS had no significant effect on the noncancerous MCF10a HMECs.

The SUM149 IBC cell line exhibited a significant increase in intracellular neutral lipid content when LDL was added to the cells. ORO staining of the IBC cells demonstrated a consistent 2.3-fold increase over cells grown in LPDS-medium and a 1.4-fold increase over the FBS control. The latter increase approached significance. After LDL addition, the MDA-MB-231 non-IBC cells displayed a dramatic 5.4-fold increase in neutral lipid staining compared to the cells grown in LPDS alone; however neutral lipid staining did not exceed that of FBS controls. In contrast to the breast cancer cells, the MCF10a HMECs showed an insignificant increase in neutral lipid content after addition of LDL. Consistent with other studies, these data suggest that MCF10a cells are capable of regulating intracellular cholesterol levels better than the two breast cancer cell lines [11, 37, 38].

### 3.2. Expression of Cholesterol Regulatory Molecules Is Different in IBC versus Non-IBC Cells

**Figure 2:**
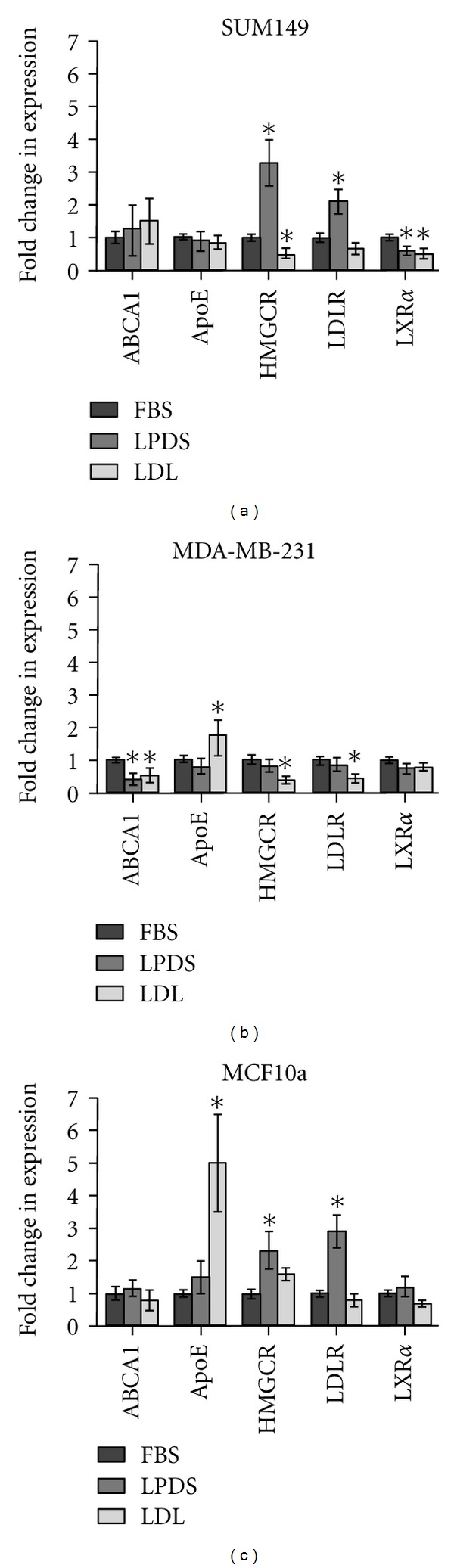
Relative mRNA fold change determined by QPCR analysis for SUM149, MDA-MB-231, and MCF10a cells. The fold-change for each gene is relative to FBS control mRNA levels. Statistical analysis was performed using one-way ANOVA, followed by Tukey's HSD test (* = *P* < 0.01). The error bars represent S.E.M. All experiments were performed at least three separate times.

To better understand the above data, we analyzed expression profiles of several transcripts associated with regulating intracellular cholesterol. Quantitative polymerase chain reaction (QPCR) was performed to determine changes in the mRNA levels of the cholesterol regulatory molecules ABCA1, ApoE, HMGCR, LDL-R, and LXR*α* that occur in each cell line after incubation with LPDS +/−LDL. As shown in Figure 2, incubation in LPDS +/−LDL resulted in significant changes in mRNA expression in a number of key genes involved in cholesterol acquisition. In the SUM149 IBC cells, mRNA expression of LDL-R significantly increased 2.2-fold, while significantly HMGCR increased 3.3-fold in LPDS-medium alone. Interestingly, both HMGCR and LDL-R expression significantly decreased in the IBC cells after the addition of LDL. Conversely, LXR*α* showed a significant 1.7-fold decrease after growth in LPDS-medium. A further 1.4-fold decrease in LXR*α* was observed with the addition of LDL, giving an overall 2.2-fold decrease in LXR*α* expression compared to the FBS control. This suggest that the SUM149 cells up-regulate HMGCR and LDL-R mRNA, but down-regulate LXR*α* mRNA when exposed to a lipoprotein deficient environment, potentially allowing them to maintain their intracellular cholesterol levels by both synthesis and uptake. These findings are consistent with the data presented from ORO staining of SUM149 cells incubated in LPDS (Figure 1), which had the smallest decrease in neutral lipid content.

In comparison to the SUM149s, the cell-type-of-origin matched, non-IBC MDA-MB-231 cell line displayed less variation in gene expression. There were no significant changes seen in ApoE, HMGCR, LDL-R, or LXR*α*, expression, after incubation in LPDS-medium. However, there was a significant 2.5-fold decrease, in ABCA1 expression. ABCA1 remained 1.9-fold lower than the FBS controls after addition of LDL. The non-IBC cells also display a significant 1.8-fold increase in ApoE expression after addition of LDL. Similarly, both HMGCR and LDL-R expression significantly decreased 2.6- and 2.3-fold, respectively, as a result of LDL treatment. Again, the lack of change in HMGCR and LDL-R in response to LPDS incubation is consistent with the ORO stains shown in Figure 1 demonstrating a dramatic decrease in intracellular lipid content. 

In contrast, the nontumorigenic MCF10a HMECs maintained relatively stable expression of ABCA1 and LXR*α* throughout LPDS and LDL treatments. When treated with LPDS, ApoE expression increased 1.8-fold compared to the FBS control. The addition of LDL resulted in a significant 5.1-fold increase in ApoE. HMGCR and LDL-R expression significantly increased 2.3- and 2.9-fold, respectively, as a result of LPDS treatment. Expression of HMGCR and LDL-R returned to levels similar to those seen in FBS control, after LDL treatment. These data suggest that the MCF10a cells respond to changes in extracellular cholesterol as cells with normal mechanisms of cholesterol regulation would [17, 18, 20, 37, 39]. Taken together, these data suggest that the breast cancer cells respond to changes in extracellular cholesterol availability in a manner that is different from immortalized mammary epithelial cells. Additionally, the IBCs respond differently to extracellular availability compared to the non-IBCs. 

**Figure 3:**
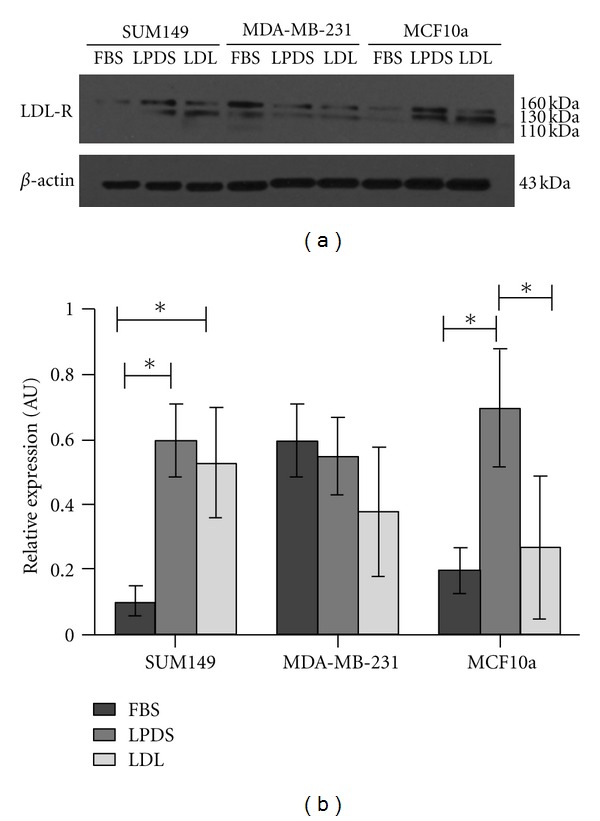
Western blot analysis of LDL-R protein expression. (a) Is a representative immunoblot for LDL-R and *β*-actin. The protein-specific bands separate at 160, 130, and at 110 kDa, according to the producer's specifications. (b) Relative LDL-R protein expression in SUM149, MDA-MB-231, and MCF10a cells as a result of FBS, LPDS, and LDL treatments. Densitometry was performed and data is presented as expression relative to *β*-Actin loading controls (*P < 0.01). Error bars represent S.E.M. and all experiments were performed at least three separate times.

Since we observed similar increases in mRNA expression of the LDL-R in the IBC and HMECs, but not the non-IBC cells grown in LPDS, we next focused on LDL-R protein expression.  Figure 3(a) is a representative immunoblot demonstrating that the changes in LDL-R protein expression after incubation in LPDS +/−LDL were consistent with changes seen in the mRNA levels as determined by QPCR analysis.  Figure 3(b) is the quantitation of relative LDL-R protein expression. These data demonstrate a significant increase of LDL-R protein due to LPDS treatment in the SUM149 and MCF10a cells. LDL-R protein levels remained significantly higher in the SUM149 cells after 12 h of treatment with LDL compared with the FBS control. Conversely in the MCF10a cells, LDL treatment significantly decreased LDL-R protein levels compared to LPDS treatment. In contrast, LDL-R protein expression was slightly decreased in the MDA-MB-231 cells as a result of LPDS and LDL treatments. We consistently observed that the SUM149 cells express the least LDL-R while the MDA-MB-231 cells have the highest expression of LDL-R in control FBS conditions.

### 3.3. Neutral Lipid Levels Effect Breast Cancer Cell Invasion

**Figure 4:**
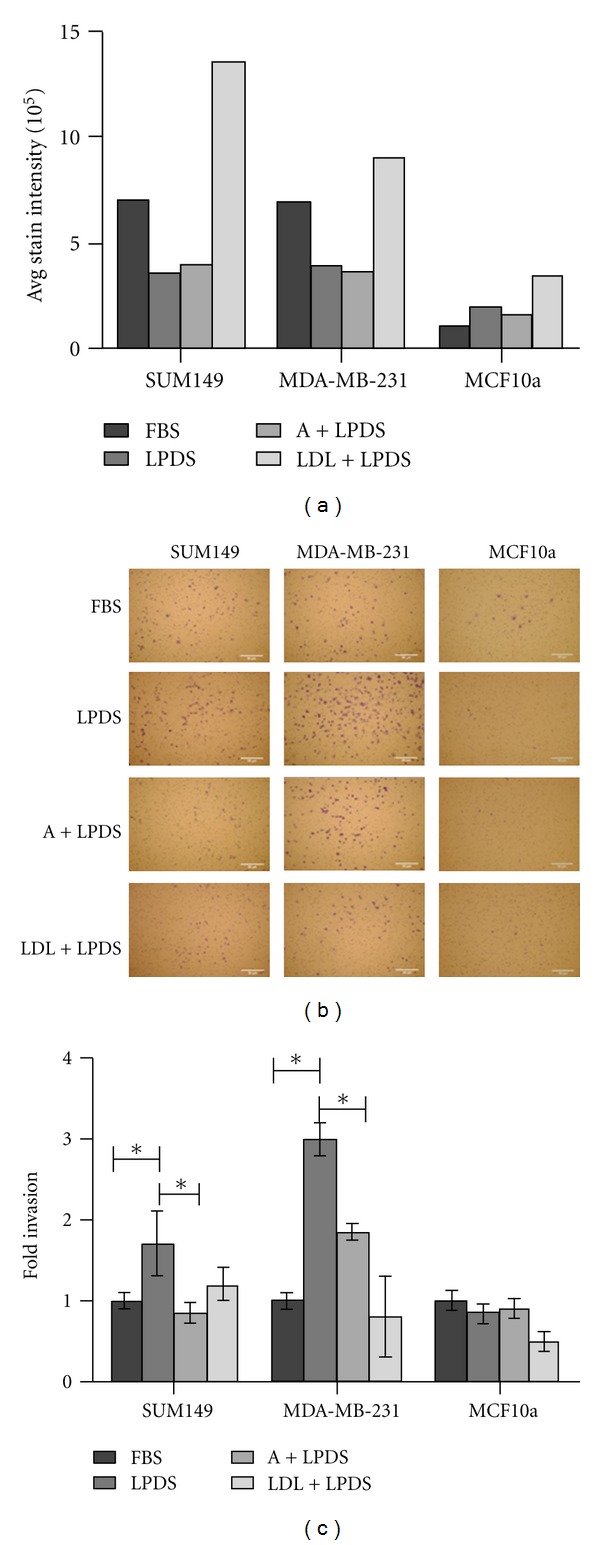
Breast cancer *in vitro* invasion assays. (a) ORO staining of treatments prior to invasion assay for SUM149, MDA-MB-231, and MCF10a cells, respectively. (b) Representative images of invasion assays. Cells were grown in medium containing FBS (untreated control), LPDS, LPDS with addition of Atorvastatin (A + LPDS) and LPDS with the addition of LDL (LDL + LPDS), resuspended in serum-free medium, placed on a Matrigel coated filter with 8 *μ* pores and allowed to invade towards a chemoattractant for 22 h. Invaded cells were stained with crystal violet and images taken using a Nikon Eclipes TE-200U. Scale bars represent 50 *μ*m. (c) Quantitation of invaded cells relative to untreated controls for SUM149, MDA-MB-231, and MCF10a cells, respectively (*P < 0.01). All experiments were performed at least three separate times.

Previous reports suggest that increased intracellular levels of cholesterol promote metastatic potential by driving invasion [13]. To explore this aspect of cholesterol storage we analyzed the effect that lipoprotein depletion and reloading has on the breast cancer cells ability to invade using an *in vitro* Matrigel invasion assay. Quantitation of a representative ORO staining of cells treated with the conditions tested in the invasion assay is shown in Figure 4(a), demonstrating the neutral lipid profile of the cells used in the invasion assay. To determine the effects of blocking *de novo* cholesterol synthesis through the mevalonate pathway, we used Atorvastatin, a commercially available HMGCR inhibitor used clinically to control cholesterol levels. Prior to the invasion assay, cells were treated with 5 *μ*M Atorvastatin in LPDS to reduce synthesis of cholesterol. As shown in Figure 4(a), SUM149 and MDA-MB-231 cells treated for 24 h with LPDS and 5 *μ*M Atorvastatin in LPDS (A + LPDS) resulted in a decrease in ORO staining intensity similar to LPDS alone. The similarity between LPDS and A + LPDS treatments is probably due to the fact that Atorvastatin blocks *de novo* synthesis of free cholesterol, which is often used in cell membranes [40, 41]. Since ORO stains neutral lipid droplets, and accumulated neutral lipids rather than the free cholesterol found in membranes, no differences between LPDS and A + LPDS is expected. Cells treated with LDL in LPDS (LDL + LPDS) displayed an increase in neutral lipid content, by ORO staining (Figure 4(a)). These data demonstrate that we were able to effectively alter neutral lipid content in each breast cancer cell line before placing them into the invasion assay. As anticipated, the MCF10a cells did not display any major difference in ORO staining after incubation in LPDS or A + LPDS. Additionally, the MCF10a cells displayed only a slight increase in ORO staining as a result of LDL + LPDS.

Invasion of the treated cells relative to untreated cells was determined. Cells were placed in serum-free medium and allowed to migrate towards serum-containing medium. Representative images of cells that have invaded through Matrigel-coated filters and the quantitation of invaded cells are provided in Figures 4(b) and 4(c), respectively. SUM149 cells in LPDS-medium showed a significant 1.7-fold increase in invasion over the control. SUM149 cells treated with A + LPDS were significantly less invasive than the LPDS-treated cells and slightly less invasive than the untreated control cells. In addition, when SUM149 cells were treated with LDL + LPDS, their invasive capacity was similar to the untreated cells. 

In comparison, MDA-MB-231 cells that were treated with LPDS were significantly 3-fold more invasive than untreated cells. Also, MDA-MB-231 cells treated with A + LPDS were twice as invasive as untreated cells, but less invasive than cells treated with LPDS alone. As seen with the SUM149 cells, treatment with LDL resulted in invasion levels similar to the FBS control. 

As expected the MCF10a cells displayed very little invasion compared to the breast cancer cells with no differences due to LPDS, A + LPDS, or LDL + LPDS. Taken together, these data suggest that cholesterol depletion increases breast cancer cell invasion towards a neutral lipid containing chemoattractant.

## 4. Discussion

Understanding the details of cholesterol uptake, storage, and metabolism in breast cancer is necessary for understanding progression. This is particularly true given that cholesterol and cholesterol-derivatives play a major role in membrane trafficking and protein localization [11, 19, 42]. We provide for the first time a comparison of cholesterol and lipid uptake and storage in IBC versus with non-IBC cells. IBC is a phenotypically and genotypically unique form of breast cancer that invades the dermal lymphatic vessels of the skin overlying the breast. Lymphatic fluid is rich in lipoproteins, containing cholesterol and other neutral lipids [43–45]. Our initial ORO staining experiments showed an abundance of visible neutral lipid droplets in both IBC and non-IBC cell lines. The presence of the neutral lipid droplets suggests accumulation of lipids, including both cholesterol and triglycerides, which was not observed in nontumorigenic, MCF10a cells. This is consistent with published high-resolution NMR studies that found malignant tissues contained greater amounts of cholesterol esters than normal breast tissues [36]. Further, Filipin staining suggests that the cholesterol in the nontumorigenic MCF10a cells is incorporated in membranes, whereas this is not seen to the same extent in the two breast cancer cell lines. 

Our data suggest that both the IBC and non-IBC cell line have an intracellular lipid content that is dependent on the extracellular environment. The SUM149 cell line appears more responsive to changes in extracellular cholesterol concentrations. The results from these experiments suggest that IBC cells have mechanisms to increase uptake and storage of extracellular cholesterol, and they exploit *de novo* synthesis of cholesterol to maintain stores of cholesterol. Conversely, we found that MDA-MB-231 cells were not capable of maintaining their intracellular cholesterol stores when placed in an environment depleted of lipoproteins. This finding suggests that MDA-MB-231 cells rely on the extracellular environment for cholesterol loading. 

It is tempting to speculate that the way these different cells handle cholesterol and lipid uptake and storage is a result of an adaptation from the physiological origin of the cancer. IBC cells invade and spread via the dermal lymphatic vessels of the skin overlying the breast. The dermal lymphatics, while abundant in cholesterol-rich lipoprotein molecules, often undergo fluctuations in composition, which are affected by diet as well as the transfer of fluids [45]. The ability of these IBC cells to maintain their cholesterol stores could potentially help them survive in the dermal lymphatic vessels. In contrast, the MDA-MB-231 cells were originally derived from a malignant pleural effusion. Cholesterol content in malignant pleural effusions is consistently high and relatively stable with an average of cholesterol concentration of 94 mg/dL [46, 47]. Our data suggests that MDA-MB-231 cells are better equipped for an environment with consistently high concentrations of cholesterol. MCF10a cells responded in a predictable manner to changes in extracellular cholesterol levels by upregulating genes that increase intracellular cholesterol when cholesterol was depleted, and upregulating genes responsible for efflux of cholesterol when cholesterol was elevated. As expected for nontumorigenic HMECs, the response of the MCF10a cells allow them to maintain stable levels of cholesterol, without collecting large stores of it in a manner similar to what is observed in normal tissues. However, these ideas will need to be tested in future experiments.

 Our invasion assay studies suggest that depletion of neutral lipids in the breast cancer cells leads to increased invasion towards a lipid-containing chemoattractant. Although FBS contains many other potential chemoattractants, we show that neutral lipid depletion by growth in LPDS leads to a significant increase in invasion. In contrast, addition of LDL results in invasion that is comparable to the untreated cells. Llaverias et al. found that malignant rat mammary metastasis increased as a result of a cholesterol-enriched diet [13]. Increasing systemic cholesterol, thereby providing higher concentrations of lipoproteins in the circulatory system were thought to increase the invasive capabilities of the mammary carcinoma. Thus, malignant cells in need of cholesterol would be more likely to invade to reach the circulatory systems. Interestingly, our results with Atorvastatin treatment of cells in LPDS demonstrate a marked difference between the IBC and non-IBC cells. In both cell lines ORO staining demonstrated a marked decrease in intracellular lipids when the cells were grown in LPDS +/−Atorvastatin, compared to FBS controls. However, a difference was consistently observed in the ability of the cells to invade. Invasion of the SUM149 cells was significantly increased when the cells were grown in LPDS. Treatment with Atorvastatin lead to a decrease in invasion comparable to the untreated controls and the LDL treated cells. In contrast, increased invasion in both the LPDS treated +/−Atorvastatin was observed for the MDA-MB-231 cells. We observed a difference in invasion for the MDA-MB-231 cells as what has been previously published [48, 49]. These previous studies demonstrated that MDA-MB-231 invasion was decreased with lipid deprivation. The main difference between our study and the former is that we measure invasion over a longer time course, 16 h versus 6 h. 

Atorvastatin inhibits the rate-limiting step of the mevalonate pathway, which is involved in *de novo* synthesis of cholesterol but also prenyl groups. Rho GTPases, including RhoC GTPase rely on prenylation to be properly localized to the plasma membrane and activated [50]. Previously, we demonstrated that active RhoC GTPase is absolutely required for IBC cell invasion (reviewed in [8, 51]). Therefore, the reduction in the Atorvastatin treated SUM149 cells may be in part due to decreased synthesis of prenyl groups and the inability of the cells in a lipid depleted environment to acquire exogenous isoprenes.

Again, it is tempting to speculate that our studies suggest that malignant breast cells, which have the propensity to accrue intracellular cholesterol, potentially seek out cholesterol by invasion when their needs are not being met in their current environment. This could provide a potential explanation of why metastatic cancer cells accumulate more neutral lipids. This would suggest that tumor cell invasion may, in part, be driven by the tumor cells need for cholesterol rather than high cellular cholesterol levels. This may have implications for the control of progression and metastasis by regulation of dietary cholesterol. Again, this is a subject for future studies.

## 5. Conclusions

Molecularly and phenotypically IBC is unique from other types of breast cancers and our findings highlight another distinct difference between these two types of breast cancers. Differences in uptake, storage, and utilization of cholesterol by these cancers could potentially be exploited therapeutically and by dietary manipulation. This is particularly relevant for IBC, which primarily resides as intralymphatic tumor emboli and lymph cholesterol concentration is directly affected by diet.

## Supplementary Material

Supplementary Figure 1: is a representative phase contrast image showing the presence of MCF10a cells in samples that were Oil Red O stained. Lack of staining makes them barely visible in the bright field image (Figure 1A). The scale bar represents 10 **μ**m.
Supplementary Figure 2: is Filipin staining of breast cancer cell lines. The stain intensity of the MCF10A, SUM149 and MDA-MB-231 cells was determined using LSM Image Examine. SUM149 and MDA-MB-231 cells display a significantly lower staining intensity than MCF10a cells (*p*<0.01, error bars are S.E.M), using One-way ANOVA followed by Tukey's HSD test (B). All experiments were performed at least three separate times.

## Figures and Tables

**Table 1:** QPCR primer sequences.

Gene	Fwd Primer	Rev Primer	Size
ABCA1	TGGCTTAGATTGGACAGCCCAAGA	AGCCAGACTTCTGTTGCTATGGGT	195
ApoE	GCCAATCACAGGCAGGAAGATGAA	ACCCAGCGCAGGTAATCCCAAA	192
B2M	TGTCTGGGTTTCATCCATCCGACA	TCACACGGCAGGCATACTCATCTT	168
HMGCR	TATGTGCTGCTTTGGCTGCATGTC	ATACCAAGGACACACAAGCTGGGA	83
LDL-R	TCAACACACAACAGCAGATGGCAC	AAGGCTAACCTGGCTGTCTAGCAA	140
LXR*α*	CATGCCTACGTCTCCATCCA	CGGAGGCTCACCAGTTTCAT	77
SR-BI	CGAGTACCGCACCTTCCAGTT	ACCAGGATGTTGGGCATGAC	81

## Abbreviations

IBC: Inflammatory breast cancer
Non-IBC: Noninflammatory breast cancer
HMEC: Human mammary epithelial cell
ORO: Oil Red O
FBS: Fetal bovine serum
LPDS: Lipoprotein deficient serum
LDL: Low-density lipoprotein
LDL-R: Low-density lipoprotein receptor
vLDL: Very low-density lipoprotein
HMGCR: 3-Hydroxy-3-methylglutaryl-CoA reductase
SR-BI: Scavenger receptor class B type I
ApoE: Apolipoprotein E
QPCR: Quantitative polymerase chain reaction.
